# Activation of Endoplasmic Reticulum-Localized Metabotropic Glutamate Receptor 5 (mGlu_5_) Triggers Calcium Release Distinct from Cell Surface Counterparts in Striatal Neurons

**DOI:** 10.3390/biom15040552

**Published:** 2025-04-09

**Authors:** Yuh-Jiin I. Jong, Steven K. Harmon, Karen L. O’Malley

**Affiliations:** Department of Neuroscience, Washington University School of Medicine, Saint Louis, MO 63110, USA; jongi@wustl.edu (Y.-J.I.J.); harmons@wustl.edu (S.K.H.)

**Keywords:** metabotropic glutamate receptor 5 (mGlu_5_), endoplasmic reticulum (ER), calcium, G protein-coupled receptor (GPCR), N-methyl-D-aspartic acid (NMDA)

## Abstract

Metabotropic glutamate receptor 5 (mGlu_5_) plays a fundamental role in synaptic plasticity, potentially serving as a therapeutic target for various neurodevelopmental and psychiatric disorders. Previously, we have shown that mGlu_5_ can also signal from intracellular membranes in the cortex, hippocampus, and striatum. Using cytoplasmic Ca^2+^ indicators, we showed that activated cell surface mGlu_5_ induced a transient Ca^2+^ increase, whereas the activation of intracellular mGlu_5_ mediated a sustained Ca^2+^ elevation in striatal neurons. Here, we used the newly designed ER-targeted Ca^2+^ sensor, ER-GCaMP6-150, as a robust, specific approach to directly monitor mGlu_5_-mediated changes in ER Ca^2+^ itself. Using this sensor, we found that the activation of cell surface mGlu_5_ led to small declines in ER Ca^2+^, whereas the activation of ER-localized mGlu_5_ resulted in rapid, more pronounced changes. The latter could be blocked by the Gq inhibitor FR9000359, the PLC inhibitor U73122, as well as IP_3_ and ryanodine receptor blockers. These data demonstrate that like cell surface and nuclear mGlu_5_, ER-localized receptors play a pivotal role in generating and shaping intracellular Ca^2+^ signals.

## 1. Introduction

As a universal signaling molecule, calcium (Ca^2+^) is a critical intracellular second messenger [1,2,3]. Cytosolic Ca^2+^ signals are generated via Ca^2+^ release from intracellular stores like the endoplasmic reticulum (ER) or via entry from the extracellular space. In the former, activation of cell surface receptors such as G protein-coupled receptors (GPCRs) or receptor tyrosine kinases (RTKs) can lead to the activation of phospholipase C (PLC), the cleavage of phosphatidyl inositol 4, 5 bisphosphate (PIP_2_) and the production of inositol 1,4,5 trisphosphate (IP_3_) [4]. IP_3_ binding to IP_3_ receptors on the ER membrane allows Ca^2+^ to be released from luminal stores, affecting many cytoplasmic signaling systems. Ca^2+^ within the ER lumen also plays a signaling role [5,6,7], affecting the ability of the ER to communicate with other organelles as well as supporting critical ER functions such as protein folding and lipid biosynthesis. Not surprisingly, disruption of ER Ca^2+^ homeostasis can lead to ER stress, loss of mitochondrial function, apoptosis, and cell death [8]. Thus, transient increases of cytoplasmic Ca^2+^ as well as perturbation of luminal Ca^2+^ can both lead to a variety of cell biological phenomena critical to cellular homeostasis.

Metabotropic glutamate receptors (mGlu receptors) are a family of GPCRs that modulate neuronal excitability and synaptic transmission in the central nervous system [9,10]. In particular, the mGlu subtype, mGlu_5_, plays a fundamental role in synaptic plasticity primarily by regulating intracellular Ca^2+^ levels via release from ER Ca^2+^ stores. Many studies have shown that activated plasma membrane mGlu_5_ couples to G_q/11_ and phosphatidylinositol (PI)-PLC to generate IP_3_-mediated release of Ca^2+^ from both IP_3_ and ryanodine intracellular receptors. Depending upon the cell type in which the receptor is expressed, activation of cell surface mGlu_5_ can generate a rapid, transient Ca^2+^ signal or a slowly decaying oscillatory response [11,12,13].

Over the last two decades, work from this lab has also shown that mGlu_5_ is one out of a growing number of GPCRs [14,15,16] that can signal from intracellular membranes [17,18,19,20,21,22]. Specifically, more than 90% of mGlu_5_ is present on intracellular membranes such as the ER and the nuclear membrane, where it can be activated by ligands that can be transported across cell membranes [17]. Like mGlu_5_ present on the cell surface, activation of endogenous mGlu_5_ receptors expressed on the inner membrane of isolated striatal nuclei also generate IP_3_, *leading to the* IP_3_-mediated release of Ca^2+^ in the nucleus [17,23]. Interestingly, in the striatum, mGlu_5_-activated nuclear response patterns yield long prolonged Ca^2+^ responses (>10 min; 17). These data showed that the nucleus could function as an autonomous organelle independent of signals originating in the cytoplasm, and that nuclear mGlu_5_ receptors play a dynamic role in mobilizing Ca^2+^ in a specific, localized fashion.

To directly monitor mGlu_5_-mediated changes in ER Ca^2+^ itself, we used the newly designed and optimized ER-targeted Ca^2+^ sensor, ER-GCaMP6-150 [24], as a robust, specific approach to bypass less interpretable changes in cytosolic Ca^2+^ versus changes in ER Ca^2+^ content. Using this tool, we found that activation of cell surface mGlu_5_ using DHPG led to a modest decline in somal ER Ca^2+^, whereas activation of intracellular mGlu_5_ using Quis resulted in more pronounced soma ER Ca^2+^ changes. In either case, the effects were almost two-fold larger when measured in neurites. ER-localized mGlu_5_-mediated Ca^2+^ responses could also be blocked by the Gq inhibitor, FR9000359, and the PLC inhibitor, U73122, but not by U73343. Finally, both IP_3_ and ryanodine receptor blockers prevented ER mGlu_5_-mediated decreases in luminal Ca^2+^. Collectively, these studies show that like cell surface and nuclear receptors, activated ER mGlu_5_ receptors couple to G_q/11_ and PLC to generate the IP_3_-mediated release of Ca^2+^ from Ca^2+^-release channels in the ER. Thus, the ER can also function as an autonomous organelle in mobilizing Ca^2+^ in a specific, localized fashion.

## 2. Materials and Methods

### 2.1. Materials

(+)-α-Amino-3,5-dioxo-1,2,4-oxadiazolidine-2-propanoic acid, quisqualate (Quis), (S)-3,5-dihydroxyphenylglycine (DHPG), 2-methyl-6-(phenylethynyl)pyridine (MPEP), 7-(hydroxyimino)-cyclopropan[b]chromen-1a-carboxylate ethyl ester (CPCCOEt), (±)-4-(4-aminophenyl)-1,2-dihydro-1-methyl-2-propylcarbamoyl-6,7-methylenedioxyphthalazine (SYM2206), 2-Aminoethoxydiphenylborane (2-APB), 1-[6-[[(17β)-3-Methoxyestra-1,3,5(10)-trien-17-yl]amino]hexyl]-1*H*-pyrrole-2,5-dione (U73122), and 1-[6-[[(17*β*)-3-Methoxyestra-1,3,5(10)-trien-17-yl]amino]hexyl]-2,5-pyrrolidinedione (U73343) were purchased from Tocris (Bio-Techne Corporation, Minneapolis, MN, USA). (4R,6S,8S,10Z,12R,14R,16E,18R,19R,20S,21S)-11,19,21-trihydroxy-4,6,8,12,14,18,20-heptamethyl-22-[(2S,2′R,5S,5′S)-octahydro-5′-[(1R)-1-hydroxyethyl]-2,5′-dimethyl [2,2′-bifuran]-5-yl]-9-oxo-10,16-docosadienoic acid (Ionomycin) and 1-[[[5-(4-nitrophenyl)-2-furanyl]methylene]amino]-2,4-imidazolidinedione, monosodium salt (Dantrolene) were purchased from Cayman Chemical (Ann Arbor, MI, USA). N-methyl-D-aspartic acid (NMDA) was purchased from Sigma-Aldrich (St. Louis, MO, USA). 2-Amino-2-(3-cis/trans-carboxycyclobutyl)-3-(9H-thioxanthen-9-yl) propionic acid (LY393053) was obtained from Lilly Research Laboratories, Eli Lilly and Company (Indianapolis, IN, USA). (3R)-N-acetyl-3-hydroxy-L-leucyl-(αR)-α-hydroxybenzenepropanoyl-2,3-didehydro-N-methylalanyl-L-alanyl-N-methyl-L-alanyl-(3R)-3-[[(2S,3R)-3-hydroxy-4-methyl-1-oxo-2-[(1-oxopropyl)amino]pentyl]oxy]-L-leucyl-N,O-dimethyl-L-threonine, (7→1)-lactone (FR900359) [25] was a gift from Dr. Ken Blumer (Washington University School of Medicine, St. Louis, MO, USA).

### 2.2. Plasmid Constructs

ER-GCaMP6-150 (Addgene plasmid #86918; 24) was a gift from Dr. Ghazaleh Ashrafi (Washington University School of Medicine) with permission from Dr. Timothy Ryan (Weill Cornell Medicine, New York, NY, USA).

### 2.3. Primary Neuronal Culture and Transfection

Primary striatal cultures using neonatal 1-day-old rat pups were prepared and maintained as previously described [17,26]. The cells were plated onto poly-d-lysine-coated, glass-inserted P35 dishes (14 mm; 60,000/glass, Cellvis, Mountain View, CA, USA) for immunostaining or Ca^2+^ real-time imaging. Cells were cultured in humidified air with 5% CO_2_ at 37 °C. The cultures were transfected with ER-GCaMP6-150 at Div (days in vitro) 11 using Lipofectamine 2000 (Thermo Fisher Scientific, Waltham, MA, USA) and immunostained or imaged in real time 4–7 days after transfection as previously described [18].

### 2.4. Immunocytochemistry

After transfection, primary striatal cultures at DIV 15–18 were fixed and stained as described previously [18]. Primary antibodies included mouse monoclonal anti-calnexin (1:50; BD Bioscience., Becton, NJ, USA) and chicken polyclonal anti-GFP (1:2000; Aves Labs., Tigard, OR, USA). Secondary antibodies included goat anti-mouse Cy3 (1:300; Jackson Immunoresearch, West Grove, PA, USA) and goat anti-chicken Alexa 488 (1:500; Molecular Probes, Eugene, OR, USA).

### 2.5. Measurement of ER Ca^2+^ Dynamics

ER-targeted, low-affinity GCaMP6-150 was used to measure ER Ca^2+^ dynamics. Transfected striatal neurons were preincubated with control medium (125 mM NaCl, 5 mM KCl, 20 mM Hepes, 1.5 mM CaCl_2_, 1.5 mM MgCl_2_, 10 mM glucose, pH 7.4) containing mGlu_1_ antagonist, CPCCOEt (20 µM), and AMPA receptor, antagonist SYM2206 (25 µM), for 30 min at 37 °C before adding DHPG (100 µM) to measure cell surface mGlu_5_ activation. In addition, the impermeable, non-transported mGlu_5_ antagonist, LY393053 (LY53, 20 µM) [17], was included in the incubation buffer before adding Quis (20 µM) to measure specific intracellular mGlu_5_ activation. To study the pathways involved in intracellular mGlu_5_ activation, U-73122 (phospholipase C inhibitor, 10 µM), U73343 (inactive analog of U73122, 10 µM), 2-APB (IP3 receptor inhibitor, 100 µM), Dantrolene (ryanodine receptor inhibitor, 10 µM), or FR900359 (G_q/11_ inhibitor,1 µM) was also included in the incubation buffer before adding Quis. In all cases, drugs at 100x concentrations were added to the side of the dish by hand-held pipette and allowed to diffuse over the cells.

### 2.6. Confocal Microscopy and Data Analysis

Fluorescent Measurements of ER Ca^2+^ were performed and quantitated as described previously [22]. Briefly, 4–7 days after transfection with GCaMP6-150, ER Ca^2+^ imaging in striatal neurons was conducted using a laser confocal microscope (Olympus BX 50WI, Center Valley, PA, USA) Fluoview1200 with an Olympus LUMPlanFl/lR 40×/0.80w objectives. Cultures were preincubated with a control medium and treated as described above. The real-time ER Ca^2+^ images were conducted at 3–5 sec intervals and collected by an Olympus Fluoview FVX Confocal Laser Scanning system using Fluoview 4.2 acquisition software (https://www.olympus-lifescience.com/en/downloads/detail-iframe/?0[downloads][id]=847249651) (accessed on 11 February 2021). Images were processed with MetaMorph (version 7.7) (https://www.moleculardevices.com/products/cellular-imaging-systems/acquisition-and-analysis-software/metamorph-microscopy) (accessed on 14 April 2023) Professional Image Analysis software, produced by Universal imaging corporation (Downingtown, PA, USA). Immunofluorescence was analyzed around the soma area or in proximal dendrites at a distance of 40 µm. The average intensity across all images in soma and neurites was calculated at each time point for each category treated with different agonists or antagonists and then compared. Fluorescent ER Ca^2+^ signals, in response to electrical activity (ΔF), were normalized to the resting fluorescence (F_0_). The F_0_ value was additionally corrected for background autofluorescence measured in a nearby non-transfected region. Separate controls were performed with each experiment, and Student’s *t* test was used to determine statistical significance. Half maximal response times (T½) were assessed from the slopes of drug-induced ER Ca^2+^ loss curves derived from the first significant point of deflection outwards to 100 s. The statistical analysis was performed by GraphPad prism 10.2 (GraphPad software, Boston, MA, USA). Aggregated data (N = 12 for each condition) were analyzed via one-way of variance (ANOVA) followed by Tukey’s multiple comparison test.

### 2.7. Animal Studies

All animal procedures were performed according to NIH guidelines and approved by the Washington University Institutional Animal Care and Use Committee under protocols 21-0052 and 22-0228. Animals were under the care of the Washington University School of Medicine Division of Comparative Medicine.

## 3. Results

**Figure 1:**
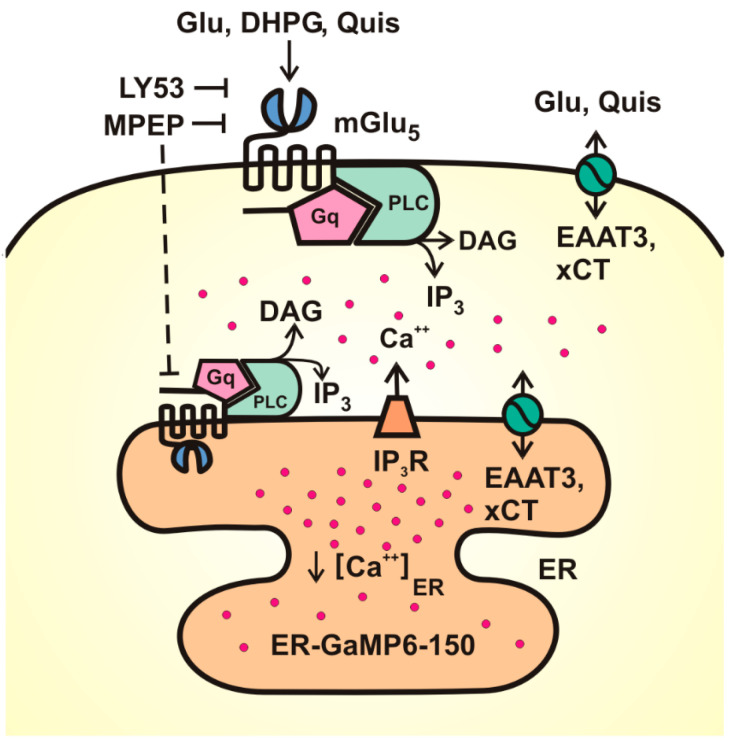
Schematic representation of cell surface and ER-localized mGlu_5_ receptor signaling in striatal neurons. Glu (glutamate) and Quis (quisqualate) act as impermeable, transported agonists and thus activate both the cell surface and intracellular mGlu_5_ receptor. DHPG is an impermeable, non-transported mGlu5 agonist; accordingly, it activates only cell surface receptors. LY53 (LY393053) is an impermeable, non-transported antagonist whereas MPEP is a permeable mGlu_5_ antagonist. Effectors downstream include Gq, phospholipase C (PLC), diacylglycerol (DAG), and IP_3_. The latter acts at IP_3_ receptors (IP_3_R) to release luminal ER Ca^2+^. This can be measured in real time via ER-GaMP6-150. EAAT3 (excitatory amino acid transporter 3) or xCT (cystine/glutamate exchanger) are bidirectional Glu or Quis transporters.

**Figure 2:**
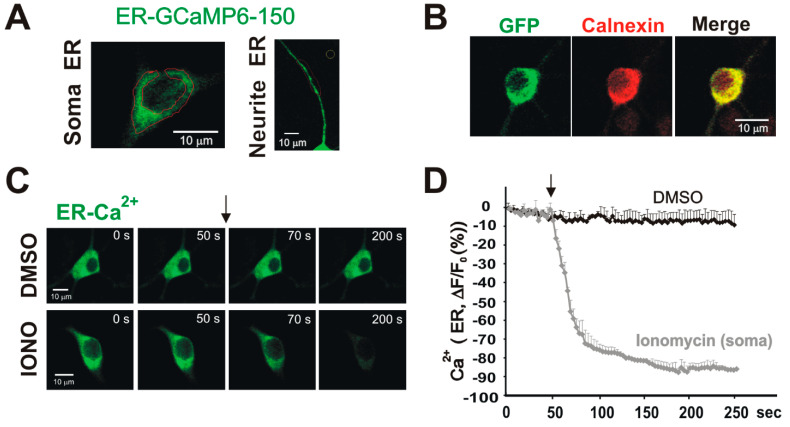
ER-GCaMP6-150 is an ER Ca^2+^ indicator. Ionomycin triggers rapid ER Ca^2+^ release in soma and neurites of striatal neurons. Striatal neurons transfected with ER-GCaMP6-150 at DIV 11 were immunostained or imaged by laser confocal microscope (Olympus BX 50WI) Fluoview1200 at DIV 15–18. (**A**) High-resolution image of striatal neurons transfected with ER-GCaMP6-150 showing ER structure in soma (left) and neurites (right). (**B**) The GCaMP6 expression is co-localized with the ER marker calnexin. (**C**) Time-lapse montages of striatal neurons transfected with ER Ca^2+^ probe (ER-GCaMP6-150) and treated with DMSO (0.04%) or ionomycin (4 µM). (**D**) Graphic quantifying changes in ER Ca^2+^ upon treatment with DMSO or ionomycin. Ionomycin induced a rapid decrease in ER Ca^2+^ (DMSO or 4 µM ionomycin was applied at the time indicated by the arrow, n = 12 for each condition).

ER-GCaMP6-150 as an ER Ca^2+^ indicator: Previously, we demonstrated that ER-localized mGlu_5_ receptors would be oriented such that the ligand-binding domain is within the ER lumen. As such, agonists must cross both the cell surface lipid bilayer as well as the ER membrane for receptor activation [17,18,23]. Mechanistically, agonist transport is achieved via the sodium-dependent glutamate transporter and/or the cystine, glutamate xCT exchanger [17,18]. Using radiolabeled glutamate and Quis, as well as uptake studies and knock-out animals, we showed that the mGlu_5_ agonist DHPG is a non-transported, non-permeable agonist, whereas glutamate and Quis enter the cell via active transport [17]. Although we see similar results in cortex, hippocampus, and spinal cord dorsal horn neurons [17,18,19,20], we typically use primary dissociated striatal neurons because >70% of the neurons are medium spiny neurons that express mGlu_5_ [17]. Inasmuch as the G protein binding portion of mGlu_5_ is in the cytoplasm, after ligand binding, G_q/11_-coupled receptors like mGlu_5_ activate PLC which, in turn, hydrolyzes the membrane PIP_2_ into the second messengers IP_3_ and diacylglycerol (DAG). IP_3_ can then activate IP_3_ receptors on the ER membrane, leading to the release of ER Ca^2+^. A schematic representation of these interactions is shown in Figure 1. The release of ER Ca^2+^ is frequently measured as an increase in cytoplasmic Ca^2+^ assessed by the Ca^2+^ indicator dye, Oregon Green^TM^ 488 BAPTA-1, AM. Alternatively, and more directly, decreases in ER Ca^2+^ content can be directly monitored by the loss of luminal fluorescence using the genetically encoded Ca^2+^ sensor, ER-GCaMP6-150 [24], that is restricted to the ER throughout the neuron (Figure 2A) and co-localizes with the ER marker calnexin (Figure 2B). Real-time imaging of somatic ER-GCaMP6-150 before and after the addition of either DMSO as a treatment control or the Ca^2+^ ionophore, ionomycin, are shown in Figure 2C and quantified in Figure 2D. Consistent with published reports using this sensor [24], ionomycin (4 µM) treatment led to a rapid decrease in ER Ca^2+^ levels (Figure 2C,D).

**Figure 3:**
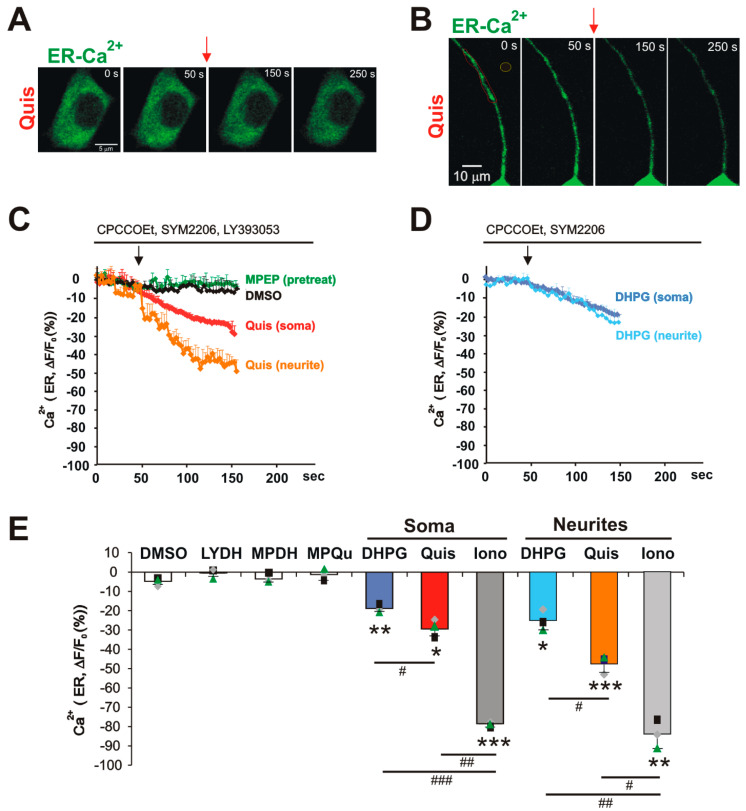
mGlu_5_ agonists trigger ER Ca^2+^ release in soma and neurites of striatal neurons. Striatal neurons transfected with ER-GCaMP6-150 and imaged as described in methods. (**A,B**) Time-lapse montages of soma (**A**) or neurites (**B**) of striatal neurons transfected with ER Ca^2+^ probe (ER-GCaMP6-150) and treated with Quis (20 µM). (**C,D**) Graphic quantifying changes in ER Ca^2+^ upon treatment with Quis (20 µM) or DHPG (100 µM). Quis or DHPG was applied at the time indicated by the arrow. (**C**) Quis triggered a pronounced ER Ca^2+^ release in soma and neurites of striatal neurons, whereas (**D**) DHPG triggered a more modest ER Ca^2+^ release. (**E**) Changes in ER Ca^2+^ 100 s after treatment with various drugs. LYDH represents LY393053 pretreatment prior to DHPG addition; MPDH refers to MPEP pretreatment prior to DHPG or Quis (MPQu). Bars represent the mean of three experiments, ±S.E.M, from 12 neurons in each condition. Individual experiments are denoted by a ▲, ■, or ♦. *, ** denotes statistical significance compared to DMSO treatment with a Student’s t test: * *p* < 0.05, ** *p* < 0.01, *** *p* < 0.001 (soma ER: *p* = 0.005 for DHPG, *p* = 0.012 for Quis, *p* = 0.0002 for ionomycin; neurite ER: *p* = 0.02 for DHPG, *p* = 0.0007 for Quis, *p* = 0.002 for ionomycin). ^#^ denotes statistical significance compared increased levels with different treatment using a Student’s t test: ^#^ *p*< 0.05, ^##^ *p* < 0.01, ^###^ *p* < 0.001 (soma ER: *p* = 0.046 for DHPG versus Quis, *p* = 0.0002 for DHPG versus ionomycin, *p* = 0.0014 for Quis versus ionomycin; neurite ER: *p* = 0.031 for DHPG versus Quis, *p* = 0.003 for DHPG versus ionomycin, *p* = 0.012 for Quis versus ionomycin).

mGlu_5_ agonists trigger ER Ca^2+^ release in soma and neurites of striatal neurons: Once the correct targeting of ER-GCaMP6-150 to the ER was verified, endogenous mGlu_5_ receptor-mediated Ca^2+^ responses were determined using pharmacological isolation of either cell surface or ER-localized receptors as we have described [18]. Consistent with the previous data, bath application of the transported agonist, Quis, generated a pronounced loss of ER Ca^2+^ in both neuronal cell bodies (−29.46 ± 4.59 S.E.M, n = 3) and neurites (−47.92 ± 4.88 S.E.M, n = 3) 100 sec after treatment (Figure 3A–C). These changes were entirely due to activation of ER-restricted mGlu_5_ since they occurred in the presence of the impermeable, non-transported antagonist, LY393053, which we have shown to block all cell surface mGlu_5_ receptor contributions [18] (Figure 3A–C). In contrast, pretreatment with the cell-permeant, mGlu_5_ antagonist, MPEP, blocked all Quis responses (Figure 3C,E). To directly assess cell surface mGlu_5_‘s ability to release ER Ca^2+^, sibling cultures transfected with ER-GCaMP5-150, were treated with the impermeable, non-transported agonist, DHPG. DHPG led to an ER Ca^2+^ loss of 18.85% ± 2.13 S.E.M, n = 3 in the soma and a loss of 25.35% ± 5.37 S.E.M, n = 3 in the neurites. Notably, DHPG had a more modest effect in triggering ER Ca^2+^ release than Quis (~66% of a Quis somal response and 55% of a Quis neurite response; Figure 3D,E). In comparison to ionomycin, which, at 100 sec, has maximally released ER Ca^2+^ levels from the soma (Figure 2D; (−78.50% ± 0.24 S.E.M, n = 3) or neurites (−83.79% ± 7.6 S.E.M, n = 3). At that same time point, DHPG released only 25–30% of ER Ca^2+^ measured in striatal soma or neurites, respectively. Quis activation of ER-localized mGlu_5_ receptors led to the release of ~40% of somal ER Ca^2+^ in comparison to ionomycin, but >50% of maximal ER Ca^2+^ release in striatal neurites (Figure 3E). Taken together, these data unequivocally establish that ER-localized mGlu_5_ is functionally active and is the major contributor to the increased cytoplasmic Ca^2+^ levels following receptor activation.

**Figure 4:**
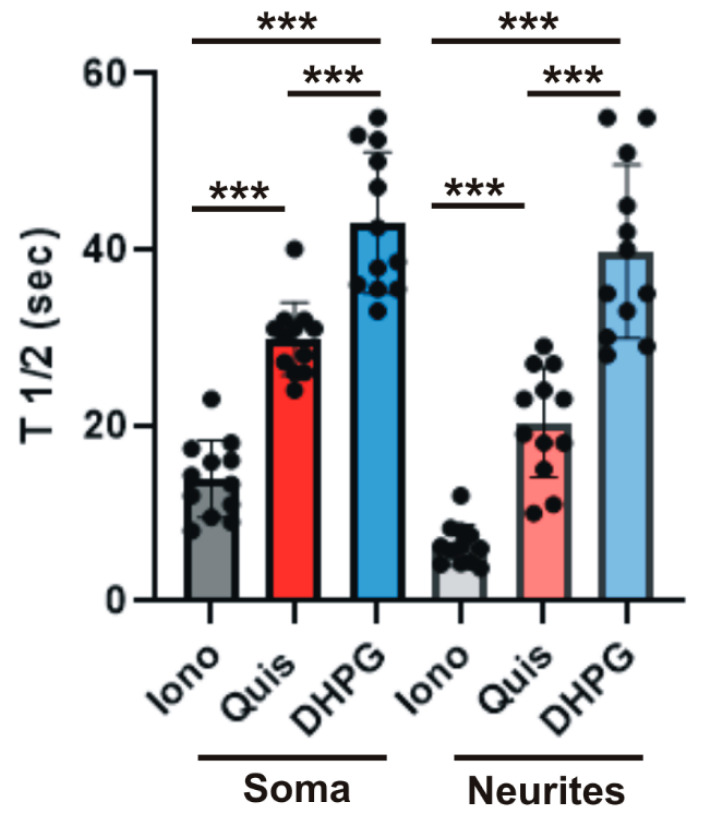
Quis triggers a more rapid ER Ca^2+^ release than DHPG in striatal neurons. Bar graphs of ER Ca^2+^ release T ½ at 100 s after drug application based on the curves in Figure 2D and Figure 3C,D. Each point represents one cell (N = 12 for each condition, error bars represent standard deviation). *** *p* < 0.001, *** denotes statistical significance between different groups. Black dots represent individual neurons. The groups were compared by one-way ANOVA followed by Tukey test.

Quis triggers more rapid ER Ca^2+^ release than DHPG in striatal neurons: Using this same preparation of dissociated striatal neurons, we previously showed that cell surface-localized and intracellular mGlu_5_ are associated with distinct patterns of Ca^2+^ release such that cell surface receptors exhibited rapid transient Ca^2+^ responses, whereas intracellular mGlu_5_ exhibited sustained Ca^2+^ signals [18,21]. Examining the slopes of ER Ca^2+^ release over time following Quis or DHPG treatment in Figure 3C,D, it is clear that Ca^2+^ is being released more rapidly following Quis application in the soma and the neurites than is the case following DHPG addition. To quantify these differences, we calculated the T½ after drug application based on experimental curves such as those represented in Figure 2D and Figure 3C,D. In keeping with the data shown in Figure 2 and Figure 3, Quis triggered a more rapid ER Ca^2+^ release than DHPG at 100 sec in striatal cell bodies (29.86 s ± 4.18 SD) and especially neurites (20.33 s ± 6.23 SD; Figure 4). Ionomycin was 13.95 s ± 4.37 SD in cell bodies and 6.40 s ± 2.32 SD in neurites. DHPG exhibited a T½ of 43.06 s ± 8.02 SD in cell bodies and 39.83 s ± 9.83 SD in neurites. In striatal neurites, Quis was ~60% of the ionomycin rate of ER Ca^2+^ release, whereas DHPG was only 30% (Figure 4). Even at 5 s, Quis was >50% of the ionomycin release rate, while DHPG was not significantly different than the DMSO control (Figure 2D and Figure 3D). Collectively, these data indicate that the activation of the ER-localized mGlu_5_ receptor releases more total ER Ca^2+^ at a faster rate than the activation of cell surface receptors.

**Figure 5:**
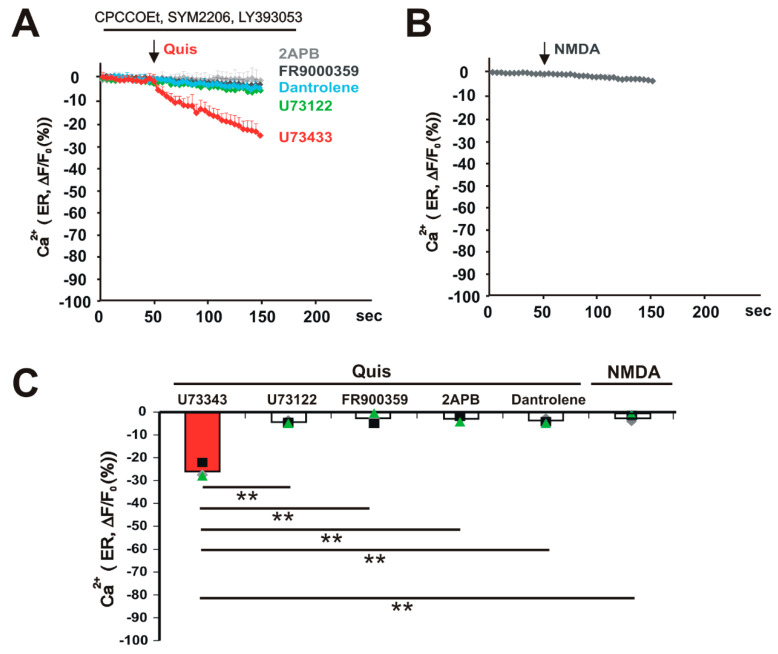
ER Ca^2+^ release triggered by Quis is blocked by U-73122, FR900359, 2-APB, and Dantrolene, whereas NMDA does not release Ca^2+^ from ER in striatal medium spiny neurons. Striatal neurons transfected with ER Ca^2+^ probe (ER-GCaMP6-150) were pretreated with U73343 (inactive analog of U73122, 10 µM), U-73122 (phospholipase C inhibitor, 10 µM), FR900359 (Gq inhibitor, 1 µM), 2-APB (inositol triphosphate receptor inhibitor, 100 µM), or Dantrolene (ryanodine receptor inhibitor, 10 µM) before applying Quis (20 µM) and imaged as described in methods. (**A**) Graphic quantifying changes in ER Ca^2+^ upon treatment with Quis (20 µM). (**B**) Graphic quantifying changes in ER Ca^2+^ upon treatment with NMDA (10 µM). (**C**) Changes in ER Ca^2+^ 100 s after applying Quis or NMDA. Bars represent the mean of three experiments, ± S.E.M, from ≥ 10 neurons in each condition. Individual experiments are denoted by a ▲, ■, or ♦. ** denotes statistical significance compared to U73343 pretreatment with a Student’s t test. ** *p* < 0.01 (For Quis-induced ER Ca^2+^ release: *p* = 0.043 for U73122, *p* = 0.0089 for FR900359, *p* = 0.017 for 2APB, *p* = 0.0043 for Dantrolene versus U73343, *p* = 0.0021 for NMDA versus Quis with U73343).

ER Ca^2+^ release triggered by Quis is blocked by U-73122, FR900359, 2-APB, and Dantrolene: ER-resident GPCRs appear to use a variety of G proteins to activate downstream pathways. In particular, release of ER Ca^2+^ has been linked to pertussis toxin-sensitive pathways, suggesting G_i/o_-driven mechanisms [27]. However, we have previously shown that, in HEK293 cells stably expressing mGlu_5_ and/or endogenous receptors expressed in striatal neurons, pertussis toxin does not affect mGlu_5_-mediated Ca^2+^ changes [23,28]. Rather, in either case, mGlu_5_ couples with G_q/11_/PLC/IP_3_ to release cytoplasmic and nucleoplasmic Ca^2+^. Similarly, we hypothesized that ER-localized mGlu_5_ couples to G_q/11_ proteins to activate downstream signaling components. To confirm whether activated ER-localized mGlu_5_ coupled to PLC to generate IP_3_-mediated release of Ca^2+^, we used the same real-time imaging assay of somatic ER-GCaMP6-150 before and after the addition of Quis in striatal neurons preincubated with various inhibitors such as 1 µM FR900359 (G_q/11_ inhibitor), 10 μM U73122 (a PLC inhibitor), 10 μM U73343 (an inactive analog of U73122), 100 μM 2-APB (an IP_3_ receptor inhibitor), and 10 µM Dantrolene (ryanodine receptor inhibitor). Results showed that ER Ca^2+^ release triggered by Quis was blocked by FR900359 (−2.74 ± 1.59 S.E.M), U-73122 (−4.37 ± 0.48 S.E.M), 2-APB (−2.94 ± 0.87 S.E.M), and Dantrolene (−3.72 ± 0.75 S.E.M). It was not blocked by U733343 (−25.85 ± 2.34 S.E.M; Figure 5A,C). These data confirm that, just like cell surface and nuclear receptors, ER-localized mGlu_5_ also couples to G_q/11_ to activate cytoplasmic PI-PLC, leading to the hydrolysis of PIP_2_ and generation of IP_3_. The latter leads to the release of Ca^2+^ from the ER into the cytoplasm. Collectively, these data show that ER-localized mGlu_5_ can function independently of signals originating at the cell surface and thus plays a dynamic role in mobilizing Ca^2+^ in a specific, localized manner.

NMDA does not release Ca^2+^ from the ER in striatal medium spiny neurons: Inasmuch as glutamate not only activates mGlu receptors but also ionotropic glutamate receptors such as NMDA, cytoplasmic Ca^2+^ changes could also be due to Ca^2+^ flux via these channels. Although we pharmacologically block these channels as well as other mGlu receptors, NMDA receptors often co-localize with metabotropic glutamate receptors to influence neuronal Ca^2+^ dynamics and synaptic plasticity [29,30,31,32,33]. To test whether NMDA affected intracellular mGlu_5_-mediated Ca^2+^ responses in striatal neurons, we quantified changes in ER Ca^2+^ upon treatment with NMDA (10 µM). Changes in ER Ca^2+^ 100 s after applying Quis or NMDA are shown in Figure 5C. Bath application of NMDA had no significant effect on ER Ca^2+^ levels (−2.29 ± 0.60 S.E.M), whereas Quis treatment of sibling cultures did (Figure 5B,C). Therefore, despite previous reports, NMDA does not influence agonist effects on intracellular mGlu_5_′s release of ER Ca^2+^.

## 4. Discussion

A growing body of data has established that GPCRs not only signal from the cell surface but also from intracellular compartments. One such receptor is mGlu_5_ which, over 20 years ago, was shown to be present and active not only on the cell surface but also on isolated nuclei [28]. Since mGlu_5_ couples to G_q/11_, these experiments used Ca^2+^ indicators such as Oregon Green BAPTA-AM or Fura2 to measure real-time changes in cytosolic Ca^2+^ levels as a proxy for release of ER Ca^2+^ [17,21]. Data from those experiments indicated that functional activity is generated by at least two separate pools of mGlu_5_—on the cell surface and on the inner nuclear membrane. The ER-GCaMP6-150 probe allowed us to directly visualize a third pool of mGlu5 receptors, namely those located on the ER, an organelle difficult to isolate in a functionally intact state. Similar to mGlu_5_ activity in purified striatal nuclei, the activation of ER-restricted mGlu_5_ produced faster, larger, and longer effects visualized with the ER-GCaMP6-150 probe than did the activation of the cell surface receptor. At peak response times, cell surface responses were ~64% of the mGlu_5_ ER response in cell bodies and 53% in neurites (Figure 3C–E). In keeping with ER peak responses being larger, the amount of Ca^2+^ released was much greater, being ~2-fold more within the cell body and ~4-fold more in neurites, measuring the area of the Ca^2+^ curve from initiation outwards to 100 s (n = 12 neurons/each condition) [20,22]. ER-restricted, mGlu_5_-mediated Ca^2+^ responses were blocked by the Gq inhibitor, FR9000359, the PLC inhibitor, U73122, as well as both IP_3_ and ryanodine receptor blockers. Thus, activated ER-restricted mGlu_5_ receptors signal via the same canonical G_q/11_/PLC/IP_3_ pathway that is found at the plasma membrane as well as the inner nuclear membrane [34,35,36,37,38,39,40]. Taken together, these data reinforce the notion that mGlu_5_ can signal from different membrane platforms to generate downstream sequelae with unique spatiotemporal profiles.

Although DHPG has been widely used to simulate a specific mGlu_5_ receptor response (e.g., “chemical” LTD in hippocampal slices) [41], it is glutamate that is released at the synapse and is transported into the cell where, as we have shown, it can activate the large intracellular pool of mGlu_5_ receptors [19]. In striatal, cortical, and spinal cord dorsal horn neurons, we have found that DHPG activation of cell surface mGlu_5_ elicited a rapid, transient Ca^2+^ response, whereas Quis (or glutamate) activation of intracellular mGlu_5_ produced sustained Ca^2+^ responses [18,20,21]. Not surprisingly, these unique spatiotemporal differences led to markedly different signaling outcomes. For example, we found that intracellular mGlu_5_ primarily uses protein synthesis-dependent MEK/ERK pathways to generate striatal LTD, whereas cell surface mGlu_5_ uses mammalian target of rapamycin complex 2 (mTORC2) [22]. Thus, it is critical to recognize that glutamate is not just activating its plethora of ionotropic and metabotropic receptors on the cell surface but, at least for mGlu_5_ and mGlu_1_, it is activating intracellular components as well.

Although generally considered to be a continuous organelle, within neurons, the ER itself is highly compartmentalized, ranging from tubules and cisternae in dendrites to sheetlike cisternae in the soma and then very narrow tubules running down the axons [42]. As the major intracellular Ca^2+^ reservoir, ER regulation of Ca^2+^ signaling is highly involved in many functions including neurotransmitter release at synapses, protein synthesis, folding, and transport throughout the neuron, especially in somatodendritic regions. ER Ca^2+^ signaling is also responsible for integrating cellular interactions with other organelles like the mitochondria and plasma membranes to maintain homeostasis [43,44,45].

Many processes have been implicated in the ER release of Ca^2+^ store regulation including Ca^2+^ entry via NMDA receptors which, in turn, can trigger ryanodine receptor activation and further ER Ca^2+^ release [29,30,31,32,33]. Activation of NMDA receptors in striatal cultures did not release Ca^2+^ from the ER (Figure 5B,C), nor did we find any evidence of ER fission in striatal neurons in which several groups have reported to be associated with NMDA-mediated ER Ca^2+^ release [33]. It has also been reported that AMPAR-associated Ca^2+^ influx might contribute to this process. Given that we included an AMPA receptor inhibitor in the culture media prior to mGlu_5_ agonist treatments, it seems doubtful that either ionic glutamate receptor is playing a role in this paradigm. Moreover, in the past, we have directly tested for AMPAR agonist effects in both wild-type and mGlu_5_ KO cultures and have seen no change in baseline Ca^2+^ levels using cytoplasmic Ca^2+^ fluorophores [18]. As an additional control in those experiments, we also tested the NMDA inhibitor, APV, and saw no effect either [18]. Thus, we concluded that neither NMDARs nor AMPARs are contributing to the release of Ca^2+^ from the ER in our dissociated striatal culture paradigm.

Taken together, all of our data are consistent with activation of intracellular mGlu_5_ directly releasing ER Ca^2+^. Collectively, these data underscore the importance of intracellular mGlu_5_ in the cascade of events underlying sustained synaptic transmission.

## 5. Conclusions

Although past experiments have used caged ligand and real-time imaging to show that intracellular mGlu_5_ is functional in dendrites, as the largest intracellular organelle, the ER extends throughout the cell, forming a complex network of tubules and sheets. Since it is impossible to isolate the entire organelle, the newly developed Ca^2+^ indicator, ER-GCaMP6-150, allows for the study of ER-localized mGlu_5_ function in situ. Using this tool, we found that activation of ER-localized mGlu_5_ resulted in pronounced somal Ca^2+^ declines compared to the cell surface receptor. Akin to the cell surface receptor, decreases in luminal Ca^2+^ were coupled to G_q/11_ and PLC to generate IP_3_-mediated release of Ca^2+^ from Ca^2+^ release channels in the ER. ER-localized mGlu_5_ effects were twice as large and more rapid than those triggered by cell surface mGlu_5_, especially in striatal neurites. Thus, the ER can function as an autonomous organelle mobilizing Ca^2+^ in a specific, localized fashion.

## Data Availability

Data used in this study are located in the article or are available upon request.
